# The impact of COVID-19 pandemic on dental practice in Iran: a questionnaire-based report

**DOI:** 10.1186/s12903-020-01341-x

**Published:** 2020-12-03

**Authors:** Hanie Ahmadi, Alireza Ebrahimi, Farhad Ghorbani

**Affiliations:** 1 Student Research Committee, Shiraz University of Medical Sciences, Shiraz, Iran; 2 Department of Oral and Maxillofacial Surgery, Shiraz University of Medical Sciences, Shiraz, Iran

**Keywords:** COVID-19, Pandemic, Dentistry, Dental practice

## Abstract

**Background:**

The highly contagious nature of the severe acute respiratory syndrome coronavirus 2 (SARS-CoV2), besides the fact that dental procedures commonly generate blood and saliva droplets that could lead to the contagion have resulted in the closure of many dental clinics. In the present study, we aimed to evaluate the impact of coronavirus disease 2019 (COVID-19) pandemic on dental practice by conducting an online questionnaire among the Iranian dental practitioners and finding their perspectives on the subject.

**Methods:**

This report is based on a questionnaire that was conducted among Iranian dentists. The survey included questions that evaluate the dentists’ perceptions and attitudes toward the COVID-19 pandemic and its effect on their personal life, financial status, and the quality of dental services for patients.

**Results:**

Overall, 240 dentists contributed to this study (214 general dentists and 26 specialists). The majority of the participants (n = 170, 70%) did not perform non-emergency procedures during the pandemic. The dental practitioners have suggested several strategies to decrease the risk of contagion, such as reducing treatment sessions (n = 90, 37%), strict triage of patients (n = 156, 64%), and using personal protective equipment (n = 108, 45%). However, most of the dentists (n = 210, 87%) had problems, providing personal protective equipment during the pandemic. Moreover, 97% (n = 234) of the participants reported that they encountered a decrease in their financial income since the eruption of the pandemic.

**Conclusion:**

Dental health care workers are at the highest risk of contracting COVID-19. Thus, dental practitioners ought to execute the standard protocols more cautiously during the pandemic. They could also lower their work hours and limit dental procedures to emergency treatments to reduce the risk of COVID-19 transmission. Besides, the public organizations should provide proper equipment for the dental practitioners to decrease the risk of contagion.

## Background

In December 2019, an outbreak of a novel beta coronavirus disease 2019 (COVID-19) began in Wuhan, China's Hubei province [1]. By now, the virus has spread all around the world and disrupted all aspects of human life. The symptoms of severe acute respiratory syndrome coronavirus 2 (SARS-CoV-2**)** are similar to the previously known coronavirus infections. These include fever, dry cough, fatigue; however, the SARS-CoV-2 has a higher spreading nature [2]. The virus could spread via respiratory droplets and contaminated surfaces, through the mucous membrane of the mouth, eyes, and nose, and even via the fecal–oral route [3, 4]. This highly contagious nature of the virus has made many medical institutions to cancel all elective procedures to reduce the risk of contagion.

The use of handpieces and ultrasonic instruments during dental procedures unavoidably results in the generation of blood and saliva droplets [5]. Consequently, these droplets could contaminate the dental instruments and the office environment. Hence, both dental practitioners and patients could be at risk of being infected with microbial pathogens [6, 7]. In this regard, researchers mentioned that dental clinics might be a possible transmission source of viruses such as human immunodeficiency virus (HIV) and hepatitis B virus (HBV); these viruses could transmit during dental practice to the patients and also the practitioners [8, 9].

The highly contagious nature of the SARS-CoV2, and the fact that dental procedures commonly generate blood and saliva droplets resulting in the spread of the virus ensued the American Dental Association (ADA) to suggest dental practitioners limit their interventions to emergency treatments [10]. Moreover, strict precautionary protocols must be performed during the pandemic to reduce the risk of infection. For instance, the dentists and their assistants should regularly provide pre-procedural mouth rinse for the patients, and frequently disinfect the dental instruments [11]. Additionally, to reduce the risk of transmission high volume saliva ejectors, anti-retraction handpieces, the rubber dam isolation, and personal protective equipment (PPE) should be used [11, 12].

The closure of dental clinics because of the COVID-19 pandemic has disrupted dental services to the patients. Moreover, the Irish Dental Association mentioned that about 75% of dental practitioners are expecting a financial loss of over 70% during the outbreak [13]. Besides, it has been mentioned that dental practitioners could be infected with the SARS-CoV-2 virus, unnoticeably**,** and become carriers of the virus [14]. As a result, the dental services were limited to the emergency and urgent cases during the early days of the pandemic [15]. In the present study, we aimed to evaluate the impact of the COVID-19 pandemic on dental practice in Iran by conducting an online questionnaire among dentists and finding their perspectives on the subject.

## Methods

### Study design and population

This report is based on a questionnaire conducted from June 10 to 25, 2020, among Iranian dentists. The total number of dentists in Iran was estimated to be 20,000, that work in public, private, and nonprofit organizations [16]. We conducted the chain-referral sampling method as most of the participants were hard to find. Our study population consists of specialists and general dentists who work in Iran regardless of their city and workplace. We asked the dentists to participate in the study via the internet (e-mail or social media) and asked them to distribute the survey among the other colleagues at their convenience. The study protocol was approved by the Medical Ethics Committee of Shiraz University of Medical Sciences. The participants have voluntarily involved in this study and written informed consent was obtained. They were assured that no personal information is required, and their filled data would be kept confidential. An online questionnaire using Google Forms was used to collect the data.

### Questionnaire

The preliminary draft of the questionnaire was designed for the present study based on experts’ opinions (5 attending dentists, Dental School, Shiraz University of Medical Sciences) and guidance from relevant literature [17, 18]. The experts above and a skilled statistician evaluated the face and content validity of the questionnaire. The content of the survey was also verified in terms of the topic concepts. Therefore, the biased, confusing, guiding, and double questions were omitted. We pilot tested the questionnaire on a sample of 20 dentists.

The survey includes 51 questions and four sections (Additional file 1) in Persian. The first section included primary demographic data. The second part consisted of closed questions (yes/no questions) about the dentists’ opinion on the COVID-19 pandemic and its effect on their personal life, financial status, and the quality of dental services for patients. The third section consists of multiple-choice questions about the dentists’ perspectives on the pandemic. The last part of the survey is based on the 5-point Likert-scale scoring to evaluate dentists' attitudes and views on the outbreak.

### Data gathering and statistical analysis

A trained person who was unaware of the names and degrees of the participants has gathered the results. Microsoft Excel sheets have been used to create tables and graphs. Two members of the research group reviewed the extracted data for accuracy. Items in the investigation were described by descriptive statistical analysis. Also, the Chi-square test was used to find any significant association between the parameters, and *P* value < 0.05 was considered as statistically significant. The calculation was performed using Statistical Package for the Social Sciences (SPSS) version 22.0 software.

## Results

Overall, 240 dentists were filled the questionnaire (214 general dentists and 26 specialists). More than half of the participants (n = 150, 62%) were between 24 to 35 years with a job experience of fewer than ten years. Among all the participants, 114 (47%) were male, 126 (52%) were female, 108 (45%) were married, and 132 (55%) were single. Moreover, 71% of the married participants had a child or more children (Table 1).

**Table 1:** The demographic data of dentists (N=240)

Patients information	Answer	Number	Percentage
Age (years)	24–35	150	62
	36–45	42	17
	46–56	42	17
	57–67	6	2
Years of experience	<10	150	62
	10–20	42	17
	20–30	42	17
	>30	6	2
Gender	Male	114	47
	Female	126	52
Marital status	Single	132	55
	Married	108	45
Number of children if married	1	49	45
	2	21	19
	3	7	6
	No children	31	29
Field of practice	Pediatric dentistry	3	1
	Prosthodontics	3	1
	Orthodontics	6	2
	Oral and Maxillofacial Radiology	3	1
	Oral and Maxillofacial Surgery	3	1
	Endodontics	3	1
	Operative dentistry	3	1
	General dentist	216	90
Health sector	Private sector	36	15
	Public sector	84	35
	Independent private clinic	120	50

Our results showed that 18 (7%) of the participants have observed the symptoms of the COVID-19 in themselves, and 3 (1%) had the disease. Besides, 9 (3%) of the participants reported that their assistants had the symptoms. Since the COVID-19 outbreak, almost one-third (n = 93, 38%) of the participants have observed an increase in their phone calls from patients for their dental problems (Table 2).

**Table 2:** The COVID-19 symptoms in dental clinics, and a rise in demand for remote consultation (N=240)

Have you experienced the following statements since the eruption of the COVID-19 pandemic?	Yes		No		Not applicable	
	Number	Percentage	Number	Percentage	Number	Percentage
A rise in phone calls from patients	93	38	84	35	63	26
Visited high-risk patients	15	6	225	93	–	–
Had symptoms of COVID-19	18	7	222	92	–	–
Had a positive test for COVID-19	3	1	12	5	225	93
Your assistants had symptoms of COVID-19	9	3	231	96	–	–
Your assistants had a positive test of COVID-19	0	0	117	48	123	51

More than half of the participants (n = 156, 64%) believed that the triage of patients should be done regarding the COVID-19 symptoms. Besides, 141 (58%) of the participants believed that the reopening of the dental clinics might increase COVID-19 incidence. Among all dentists, more than half of them (n = 162, 66%) believed that dental practice standards would reform because of the pandemic (Table 3).

**Table 3:** Dentists’ viewpoints regarding the effects of COVID-19 on dental practice (N=240)

How do you agree/disagree with the following statements?	Completely agree		Agree		Somewhat agree		Disagree		Completely disagree	
	Number	Percentage	Number	Percentage	Number	Percentage	Number	Percentage	Number	Percentage
Consulting via phone call is effective for resolving the patients’ problems	3	1	15	6	69	28	81	33	72	30
Examining the patient for COVID-19 symptoms is an essential task	105	43	51	21	42	17	30	12	12	5
Taking COVID-19 test for patients should be a routine	105	43	57	23	36	15	51	6	27	11
Reopening of the dental clinics will lead to the virus spread	81	33	60	25	51	21	15	6	33	13
Dental practice is being continued regardless of the stage of the COVID-19 pandemic	12	5	33	13	72	30	63	26	60	25
I have been facing financial problems because of the pandemic	105	43	51	21	48	20	15	6	21	8
I will face financial problems soon because of the pandemic	90	37	96	40	30	12	15	6	9	3
I had the symptoms of anxiety and depression during these times	54	22	51	21	45	18	45	18	45	18
I feel that I need to consult with a psychiatrist	21	8	75	31	39	16	39	16	66	27
I have been following the latest news about the COVID-19	102	42	78	32	36	15	18	7	6	21
Following the latest news of pandemic have been useful to me	36	15	57	23	84	351	42	7	21	8
Following the latest news cause depression and anxiety	45	18	63	26	51	21	63	26	18	7
The published guidelines for dental practice during COVID-19 are helpful	33	13	81	33	99	41	24	10	3	1
The guidelines for dental practice during COVID-19 will be changed in the future	45	18	117	48	57	23	21	8	0	0
Using PPE could effectively prevent the virus transmission	57	23	84	35	60	251	30	2	9	3

The majority of the participants (n = 170, 70%) did not perform non-emergency procedures during the pandemic, and 228 (95%) of them had changed their work hours. The contributors have had different concepts regarding the changes that are needed to be made in dental practice standards; as 207 (86%) focus on preventive care, not perform unnecessary treatments and reduce the treatment sessions at the lowest possible, in the future (Table 4).

**Table 4:** Dentists reconciled their practice to the pandemic (N=240)

How have you coped with the disruption that is caused by the COVID-19 pandemic?	Yes		No	
	Number	Percentage	Number	Percentage
I did not change my work hours, and I have been performing non-emergency procedures for the patients due to the financial reasons	70	29	170	70
I changed my work hours, and I limited the practice to the urgent and emergent cases	228	95	12	5
The dental practice standards should be changed to emphasize preventive care, not perform unnecessary treatments and reduce the treatment sessions	207	86	33	14
I have been following the latest published guidelines for dental practice during the pandemic	195	81	45	18
I have been implementing the latest guidelines during the practice	204	85	36	15

Additionally, 111 (46%) of the participants said that they had canceled all dental procedures temporarily, since the outbreak. Furthermore, almost half of the contributors (n = 117, 48%) believed that dental clinics should be closed until the end of the pandemic (Table 5). No significant association was found between the dentists’ job experience and their attitude regarding the closure of the dental clinics (*P* value > 0.05).

**Table 5:** Dentists’ experiences during the pandemic (N=240)

Question	Answer	Number	Percentage
How have you changed your treatment plans during the COVID-19 pandemic?	Nothing has changed	3	1
	Canceled all treatments until the end of the pandemic	63	26
	Canceled all treatment until the end of the alert phase of the pandemic	111	46
	Performed emergency treatment	63	26
What kind of non-emergency procedure should you do during the pandemic?	Do not perform any non-emergency treatment	198	82
	Aesthetic dental procedures	6	2
	Restorative treatment of asymptomatic caries lesion	3	1
	Extraction of asymptomatic teeth	6	2
	Initial examination	27	11
When the dental clinics should revive their normal work hour?	Until the end of the COVID-19 pandemic	117	48
	Till the end of the alert phase	111	46
	The clinic should be open now	12	5
What is your strategy of choice regarding the reopening of dental clinics?	I do not intend to work until the end of the COVID-19 pandemic	72	30
	Visiting patients who don’t have COVID-19 symptoms	21	8
	Taking COVID-19 test for patients	39	16
	Using PPE	108	45
Should you have more free time these days, how do you fill the time?	Do not have free time	15	6
	Do research	15	6
	Communicate with others	27	11
	Study	144	60
	Do exercise	39	16
Which of the following equipment has been a scarce item during the pandemic?	I have not had a problem finding PPE	9	3
	Disinfectant solutions	24	10

Finding and providing PPE was a problem for most of the dentists (n = 210, 87%), during the outbreak. About all of the participants reported that they had to buy PPE with a considerably higher price (n = 234, 98%) (Table 6). We did not find any significant association between the consumption of PPE and dentists’ job experience (*P* value > 0.05).

**Table 6:** Dentists have encountered several problems because of the pandemic (N=240)

Eruption? Did you receive any help in overcoming these difficulties?	Yes		No		Not applicable	
	Number	Percentage	Number	Percentage	Number	Percentage
Finding PPE	210	87	30	12	–	–
Rising in the price of PPE	234	98	6	2	–	–
Received help from public organizations for providing PPE	27	11	213	88	–	–
The consumption of PPE has been increased	234	97	6	2	–	–
A decrease in income	234	97	6	2	–	–
Received financial help from public organizations	6	2	231	96	3	1
Used another source of income except dental practice for daily expenditure	90	37	150	62	–	–
Encountered with financial problems in the future	138	57	102	42	–	–
Dismissed the assistants because of financial problems	105	43	135	56	–	–
The assistants decided not to work during the pandemic	99	41	141	58	–	–
Had to pay the assistants’ salary regardless of the closure of dental clinics	126	52	48	20	66	27
Recommended your assistants for getting help from unemployment insurances	57	23	183	76	–	–

Most of the participants (n = 234, 97%) reported that they encountered a decrease in their financial income since the eruption of the pandemic, while only 6 (2%) of them received financial help from public organizations. More than one-third of them (n = 90, 37%) needed another source of income for daily expenditure. No significant association was found between the decrease in financial income and dentists’ job experience or marital status (*P* value > 0.05).

## Discussion

The results of our study showed that about 7% of Iranian dentists had experienced the symptoms of COVID-19, and nearly 1% of them had a positive COVID-19 test. Besides, the workers in dental clinics are also at considerable risk of contagion, as our investigation showed that 3% of the contributors’ assistants had the symptoms mentioned above. This indicates that dental practice should be done even with more infection control cautionary, and the non-emergency treatments should be delayed until the end of the pandemic [19].

The nosocomial transmission of SARS-CoV-2 has been a concern for dental practitioners, as it could put both patients and dentists at the risk of contagion [20]. As previous studies also mentioned that dental practitioners are at higher risk of being infected by SARS-CoV-2 [21–23].

The majority of the participants of the present study reported a tremendous increase in the demand for remote dental consultations. However, they did not consider remote consultation as an effective way of delivering dental services. We believe these results could be because of the characteristics of dental procedures and the lack of appropriate infrastructure. Future studies must be conducted to hypothesize and design advanced technologies that can virtually deliver dental services [24].

Occupational Safety and Health Administration has mentioned that using remote dental consultations should be considered for the non-emergent cases during the pandemic [25]. Additionally, before the current pandemic, remote consultation was found to have sufficient quality for oral treatments [26]. The telehealth-based delivery of dental services seems to be an attractive and flexible concept, especially during these unprecedented times [27]. Despite this, most clinics do not have the proper equipment such as network infrastructures and adequately trained staff to provide telehealth services [23].

A significant number of the participants mentioned that they do not perform any non-emergency procedures, and they have lowered their work hours to minimize the spread of the virus. They also declared that they follow and implement the latest national and international COVID-19 guidelines for dental practice. However, more than half of them believed that the standards in that regard must be reformed by the local authorities. We believe that comprehensive worldwide instruction must be provided for dental settings to minimize the risk of infection, effectively.

In response to the current pandemic, several organizations such as the Centers for Disease Control and Prevention (CDC), ADA, British Dental Association, and National Health Service have designed and developed response groups, and guidance for dental settings [10, 28, 29]. These instructions emphasized on closely examining the patients considering the clinical symptoms and epidemiological history [23]. In the early days of the pandemic, the guidelines also recommended that dental care procedures should be done for urgent and emergency diagnosis while providing appropriate PPE and patient care supplies [4].

In our study, several suggestions have been made to decrease the risk of infection such as reducing the treatment sessions, emphasis on preventive care, triaging patients for the related symptoms, conducting COVID-19 tests for the referred patients, and proper use of PPE. Some participants believed that the reopening of the dental clinics for non-emergency cases might increase COVID-19 incidence, and the offices should be closed until the end of the pandemic.

The public organizations suggested that the general population should increase their oral hygiene and implement preventive care to reduce the need for dental procedures, during the pandemic [23]. As most of the dental clinics only provide low-risk procedures such as tooth extraction, which could increase the demand for removable prosthetic treatments in the future [30]. However, as the pandemic continued, it has been proposed that dental settings can deliver non-emergency treatments as well. A survey led by the ADA Health Policy Institute demonstrated that over 90% of dental clinics are now open for elective care services [31]. CDC has also designed a standard for health-care systems and health-care workers for the delivery of non-emergent services to minimize the risk of contagion [32].

Effective use of PPE, including, gowns, gloves, face shields, goggles, and face masks, is an essential regulation for preventing the spread of the virus to and from health-care providers and patients [33, 34]. While the rapid enhance of demand for PPE resulted in the shortage of these supplies all around the world [35]. The majority of the participants of the present study have asserted that the consumption of PPE had been significantly raised, and more than half of them had trouble finding facemask since the COVID-19 outbreak. Furthermore, they reported that the price of PPE had been significantly raised, which could be a sign of shortage. This increasing price of PPE might also lead to the rise of dental treatment costs [23]. Although, public organizations did not help the participants to provide this equipment.

A significant number of the participants had financial problems caused by their lowered work hours and restricted dental procedures. Consistently, a study revealed that the COVID-19 pandemic imposed financial distress on dental offices [36]. More than half of the Iranian dentists have been expending their saves for daily expenditure. Still, a small number of them have received financial help from public organizations. These results indicate that the related organizations must increase their efforts to fund the dentists and their assistants during these unprecedented times. Should not providing the support funds for the dental care workers, by persisting the COVID-19 pandemic, the number of workers that encounter financial problems will increase [23].

Our study also showed that about half of the participants had symptoms of depression and anxiety. It has been noted that the health-care workers are encountering far more emotional stress compare with the general population, during the COVID-19 pandemic [37, 38]. Increased workload, working with repeatedly changing protocols, using PPE, social-distancing, self-isolation, and caring for deteriorating patients are found to be the main concerns among the medical staff during the pandemics [39, 40]. Moreover, difficult decisions should be made by the workers during the pandemics as the resources are limited [41]. The dentists who participated in the present study also mentioned that they need to consult with a psychiatrist or a therapist.

Our study has some limitations. One of the most important weaknesses of our study is the sampling method. Although chain-referral sampling is an easy and quick method to find participants, people may refuse to participate in the study after the invitation. Furthermore, participants may recommend a dentist whom they know with a similar age range. Our investigation is a descriptive study that focuses on descriptive analysis of the situation and objects; therefore, it was unable to test or verify the causal relationship. Another weakness of our study is that we had a limited time frame to conduct the questionnaires to be more up to date.

## Conclusion

Most of the Iranian dentists have followed the latest COVID-19 guidelines. Besides, they preferred to lower their work hours and limit dental procedures to emergency treatments until the end of the pandemic. They also believed that the full reopening of the dental clinics might lead to an increase in the COVID-19 transmission. Moreover, the dentists encountered financial problems because of the closure of dental clinics. Besides, depression and anxiety were common symptoms among dentists during these times. We believe that the public organizations must intervene to financially and psychologically support the dentists during these unprecedented times. Researchers must also take reasonable steps to evaluate the impacts of COVID-19 on dental practice to find solutions that can be used during the current and future pandemics.

## Supplementary information

**Additional file 1.** The questionnaire that was used to evaluate the impacts of COVID-19 on dental practice in Iran.

## Data Availability

The datasets used and/or analyzed during the current study are available from the corresponding author on reasonable request.

## Abbreviations

ADA: American Dental Association
CDC: Centers for Disease Control and Prevention
COVID-19: Coronavirus disease 2019
HBV: Hepatitis B virus
HIV: Human immunodeficiency virus
PPE: Personal protective equipment
SARS-CoV-2: Severe acute respiratory syndrome coronavirus 2

## References

[1] Yang W, Sirajuddin A, Zhang X, Liu G, Teng Z, Zhao S, Lu M. The role of imaging in 2019 novel coronavirus pneumonia (COVID-19). European Radiology. 2020. 10.1007/s00330-020-06827-4. 10.1007/s00330-020-06827-4 PMC7156903 32296940

[2] Wu D, Wu T, Liu Q, Yang Z (2020). The SARS-CoV-2 outbreak: what we know. Int J Infect Dis.

[3] Guo YR, Cao QD, Hong ZS (2020). The origin, transmission and clinical therapies on coronavirus disease 2019 (COVID-19) outbreak – an update on the status. Military Med Res.

[4] Peng X, Xu X, Li Y, Cheng L, Zhou X, Ren B (2020). Transmission routes of 2019-nCoV and controls in dental practice. Int J Oral Sci.

[5] Kohn WG, Harte JA, Malvitz DM, Collins AS, Cleveland JL, Eklund KJ, Guidelines for infection control in dental health care settings - 2003. Journal of the American Dental Association. American Dental Association. 2004. 10.14219/jada.archive.2004.0019 14959873

[6] Bolyard EA, Tablan OC, Williams WW, Pearson ML, Shapiro CN, Deitchman SD (1998). Guideline for infection control in healthcare personnel, 1998. Infect Control Hosp Epidemiol.

[7] To KK-W, Tsang OT-Y, Yip CC-Y, Chan K-H, Wu T-C, Chan JM-C (2020). Consistent detection of 2019 Novel coronavirus in saliva. Clin Infect Dis.

[8] Updated U (2001). Public Health Service guidelines for the management of occupational exposures to HBV, HCV, and HIV and recommendations for postexposure prophylaxis. MMWR Recomm Rep.

[9] Ippolito G, Puro V, Heptonstall J, Jagger J, De Carli G, Petrosillo N (1999). Occupational human immunodeficiency virus infection in health care workers: worldwide cases through September 1997. Clin Infect Dis.

[10] ADA. Return to Work Interim Guidance Toolkit.: American Dental Association; 2020 [cited 2020 July 23, 2020]. https://success.ada.org/~/media/CPS/Files/Open%20Files/ADA_Return_to_Work_Toolkit.pdf.

[11] CDC. Guidance for Dental Settings: Centers for Disease Control and Prevention; June 17, 2020. https://www.cdc.gov/coronavirus/2019-ncov/hcp/dental-settings.html.

[12] Villani FA, Aiuto R, Paglia L, Re D (2020). COVID-19 and dentistry: prevention in dental practice, a literature review. Int J Environ Res Public Health.

[13] Farooq I, Ali S. COVID-19 outbreak and its monetary implications for dental practices, hospitals, and healthcare workers. Postgrad Med J. 2020:postgradmedj-2020–137781. 10.1136/postgradmedj-2020-137781 PMC10016845 32245754

[14] Wax RS, Christian MD (2020). Practical recommendations for critical care and anesthesiology teams caring for novel coronavirus (2019-nCoV) patients. Can J Anesth/J Can Anesth.

[15] Guo H, Zhou Y, Liu X, Tan J. The impact of the COVID-19 epidemic on the utilization of emergency dental services. J Dent Sci. 2020. 10.1016/j.jds.2020.02.002. 10.1016/j.jds.2020.02.002 PMC7156222 32296495

[16] Rezaei S, Hajizadeh M, Irandoost SF, Salimi Y (2019). Socioeconomic inequality in dental care utilization in Iran: a decomposition approach. Int J Equity Health.

[17] Geldsetzer P (2020). Knowledge and perceptions of COVID-19 among the general public in the United States and the United Kingdom: a cross-sectional Online Survey. Ann Intern Med.

[18] Khader Y, Al Nsour M, Al-Batayneh OB, Saadeh R, Bashier H, Alfaqih M (2020). Dentists' awareness, perception, and attitude regarding COVID-19 and infection control: cross-sectional study among Jordanian Dentists. JMIR Public Health Surveill.

[19] Checchi V, Bellini P, Bencivenni D, Consolo U. COVID‐19 dentistry‐related aspects: a literature overview. Int Dent J. 2020. 10.1111/idj.12601. 10.1111/idj.12601 PMC7361251 33616049

[20] Rothe C, Schunk M, Sothmann P, Bretzel G, Froeschl G, Wallrauch C (2020). Transmission of 2019-nCoV infection from an asymptomatic contact in Germany. N Engl J Med.

[21] Meng L, Hua F, Bian Z (2020). Coronavirus disease 2019 (COVID-19): emerging and future challenges for dental and oral medicine. J Dent Res.

[22] Jamal M, Shah M, Almarzooqi SH (2020). Overview of transnational recommendations for COVID-19 transmission control in dental care settings. Oral Dis..

[23] Barabari P, Moharamzadeh K (2020). Novel Coronavirus (COVID-19) and Dentistry-A Comprehensive Review of Literature. Dent J (Basel).

[24] Ebrahimi A, Ebrahimi S, Ashkani ES (2020). How COVID-19 pandemic can lead to promotion of remote medical education and democratization of education?. J Adv Med Educ Prof.

[25] OSHA. COVID-19 Guidance for dental practitioners: occupational safety and health administration; May 2020 [cited 2020 June 2020]. https://www.osha.gov/Publications/OSHA4019.pdf.

[26] Ignatius E, Perälä S, Mäkelä K (2010). Use of videoconferencing for consultation in dental prosthetics and oral rehabilitation. J Telemed Telecare.

[27] Volgenant CMC, Persoon IF, de Ruijter RAG, de Soet JJ(H). Infection control in dental health care during and after the SARS‐CoV‐2 outbreak. Oral Dis. 2020;00:1–10. 10.1111/odi.13408 PMC7272817 32391651

[28] CDC. Guidance for Dental Settings: Centers for Disease Control and Prevention; 2020 [cited 2020 Aug. 28, 2020]. https://www.cdc.gov/coronavirus/2019-ncov/hcp/dental-settings.html.

[29] BDA. Coronavirus and dentistry: British Dental Association; 2020 [cited 2020 september 11, 2020]. https://bda.org/advice/Coronavirus/Pages/latest-updates.aspx.

[30] Ali Z, Baker S, Barabari P, Martin N (2017). Efficacy of removable partial denture treatment: a retrospective oral health-related quality of life evaluation. Eur J Prosthodont Restor Dent.

[31] ADA. HPI polling shows robust, sustained rebound in dental care: American Dental Association Health Policy Institute June 05, 2020 [June 2020]. https://www.ada.org/en/publications/ada-news/2020-archive/june/hpi-polling-shows-robust-sustained-rebound-in-dental-care?&utm_source=cpsorg&utm_medium=covid-main-lp&utm_content=cv-hpi-view-poll-results&utm_campaign=covid-19.

[32] CDC. Framework for Healthcare Systems Providing Non-COVID-19 Clinical Care During the COVID-19 Pandemic: Centers for Disease Control and Prevention May 12, 2020. https://www.cdc.gov/coronavirus/2019-ncov/hcp/framework-non-COVID-care.html.

[33] Sarkarat F, Tootoonchian A, Haraji A, Rastegarmoghaddam Shaldoozi H, Mostafavi M, Naghibi Sistani SMM (2020). Evaluation of dentistry staff involvement with COVID-19 in the first 3 month of epidemiologic spreading in Iran. J Res Dent Sci..

[34] Arellano-Cotrina JJ, Marengo-Coronel N, Atoche-Socola KJ, Peña-Soto C, Arriola-Guillén LE. Effectiveness and recommendations for the use of dental masks in the prevention of COVID-19: a literature review. Disaster Med Public Health Preparedness. 2020:1–6. 10.1017/dmp.2020.255 PMC7411468 32674741

[35] Cook TM (2020). Personal protective equipment during the coronavirus disease (COVID) 2019 pandemic – a narrative review. Anaesthesia.

[36] Schwendicke F, Krois J, Gomez J (2020). Impact of SARS-CoV2 (Covid-19) on dental practices: economic analysis. J Dent..

[37] Greenberg N, Docherty M, Gnanapragasam S, Wessely S (2020). Managing mental health challenges faced by healthcare workers during covid-19 pandemic. BMJ..

[38] Chew NW, Lee GK, Jing M (2020). Psychological impact of the COVID-19 pandemic on health care workers in Singapore. Ann Intern Med..

[39] Dai Y, Hu G, Xiong H, Qiu H, Yuan X. Psychological impact of the coronavirus disease 2019 (COVID-19) outbreak on healthcare workers in China. MedRxiv. 2020 Jan 1.

[40] Dong Z-Q, Ma J, Hao Y-N, Shen X-L, Liu F, Gao Y (2020). The social psychological impact of the COVID-19 pandemic on medical staff in China: a cross-sectional study. Eur Psychiatry.

[41] Kirkpatrick JN, Hull SC, Fedson S, Mullen B, Goodlin SJ (2020). Scarce-resource allocation and patient triage during the COVID-19 pandemic. J Am Coll Cardiol.

