# Supplementary material for: The impact of COVID-19 pandemic on dental practice in Iran: a questionnaire-based report
Source: BMC Oral Health. 2020 Dec 3;20:354. doi: 10.1186/s12903-020-01341-x (PMC7711254; doi:10.1186/s12903-020-01341-x)
Supplement: Supplementary file 1 — Additional file 1. The questionnaire that was used to evaluate the impacts of COVID-19 on dental practice in Iran. [file 12903_2020_1341_MOESM1_ESM.docx]

**Questionnaire form**

**Consent**

The following consent form was prepared by the author for field methods classes, based on questions discussed about establishing informed consent.

I, ............................................., agree to participate in the investigation *“The Impact of COVID-19 Pandemic on Dental Practice: A questionnaire-based report*” that conducted by Department of Oral and Maxillofacial Surgery, Shiraz University of Medical Sciences under the supervision of Dr. Farhad Ghorbani from June 10 to 25.

**Background**

The highly contagious nature of the novel coronavirus, besides the fact that dental procedures commonly generate blood and saliva droplets that are the routes of contagion lead to the closure of many dental clinics. In the present study, we aimed to evaluate the impact of coronavirus disease 2019 (COVID-19) pandemic on dental practice by conducting an online questionnaire. The questionnaire includes 51 questions and 4 sections.

We appreciate your time and participation.

**Section one**

| 1 | Age (years) | 57-67 |
| --- | --- | --- |
|  |  | 46-56 |
|  |  | 36-45 |
|  |  | 24-35 |
| 2 | Years of experience | >30 |
|  |  | 20-30 |
|  |  | 10-20 |
|  |  | <10 |
| 3 | Gender | Male |
|  |  | Female |
| 4 | Marital Status | Single |
|  |  | Married |
| 5 | Number of children if married | 3 |
|  |  | 2 |
|  |  | 1 |
|  |  | No children |
| 6 | Field of practice | Pediatric Dentistry |
|  |  | Prosthodontics |
|  |  | Orthodontics |
|  |  | Oral and Maxillofacial Radiology |
|  |  | Oral and Maxillofacial Surgery |
|  |  | Endodontics |
|  |  | Operative Dentistry |
|  |  | General Dentist |
| 7 | Health sector | Private sector |
|  |  | Public sector |
|  |  | Independent Private clinic |

**Section two**

|  | Have you experienced the following statements since the eruption of the COVID-19 pandemic? | Yes | No | Not applicable |
| --- | --- | --- | --- | --- |
| 8 | A rise in phone calls from patients |  |  |  |
| 9 | Visited high-risk patients |  |  |  |
| 10 | Had symptoms of COVID-19 |  |  |  |
| 11 | Had a positive test for COVID-19 |  |  |  |
| 12 | Your assistants had symptoms of COVID-19 |  |  |  |
| 13 | Your assistants had a positive test of COVID-19 |  |  |  |
| 14 | Changed in working time and dental practice |  |  |  |
| 15 | Performed non-emergency procedures | Because of the patients’ request |  |  |
|  |  | Because of financial problems |  |  |
| 16 | Changed the dental practice standards | Focus on preventive care |  |  |
|  |  | Not performing unnecessary treatment |  |  |
|  |  | Reducing the treatment sessions |  |  |
| 17 | Reviewed the latest guidelines towards the COVID-19 pandemic |  |  |  |
| 18 | Implemented the latest guidelines about doing dental procedures during the COVID-19 pandemic |  |  |  |
| 19 | Had problem with providing personal protective equipment (PPE) |  |  |  |
| 20 | Rising in the price of PPE | More than 100% |  |  |
|  |  | Between 75% to 100% |  |  |
|  |  | Between 50% to 75% |  |  |
|  |  | Between 25% to 50% |  |  |
|  |  | Up to 25% |  |  |
| 21 | Received help from a public organization for providing PPE |  |  |  |
| 22 | Increased in the consumption of PPE while performing dental procedures |  |  |  |
| 23 | A decrease in income |  |  |  |
| 24 | Received financial help from a public organization |  |  |  |
| 25 | Used another source of income for daily expenditure |  |  |  |
| 26 | Encountered with financial problems |  | Up to 2 months |  |
|  |  |  | Up to 6 months |  |
|  |  |  | Up to a year |  |
|  |  |  | Never |  |
| 27 | Dismissed your assistants because of financial problems |  |  |  |
| 28 | Your assistants decided not to work during COVID-19 pandemic |  |  |  |
| 29 | Paid assistants’ salary regardless of the closure of dental clinics |  |  |  |
| 30 | Recommended your assistants for getting help from unemployment insurances |  |  |  |

**Section three**

| 31 | How have you changed your treatment plans during the COVID-19 pandemic? | Nothing has changed |
| --- | --- | --- |
|  |  | Canceled all treatments until the end of the pandemic |
|  |  | Canceled all treatment until the end of the alert phase of the pandemic |
|  |  | Performed emergency treatment |
| 32 | What kind of non-emergency treatments should you do during the pandemic?  ­­­ | Do not perform any non-emergency treatment |
|  |  | Aesthetic dental procedures |
|  |  | Restorative treatment of asymptomatic caries lesion |
|  |  | Extraction of asymptomatic teeth |
|  |  | Initial examination |
| 33 | When the dental clinics should revive their normal work hour? | Until the end of the COVID-19 pandemic |
|  |  | Till the end of the alert phase |
|  |  | The clinic should be open now |
| 34 | What is your strategy of choice regarding the reopening of dental clinics?  ­ | I do not intend to work until the end of COVID-19 pandemic |
|  |  | Visiting patients who don’t have COVID-19 symptoms |
|  |  | Taking COVID-19 test for patients |
|  |  | Using PPE |
| 35 | Should you have more free time these days, how do you fill the time? | Do not have free time |
|  |  | Do research |
|  |  | Communicate with others |
|  |  | Study |
|  |  | Do exercise |
| 36 | Which of the following equipment has been a scarce item during the pandemic? | I have not had a problem finding PPE |
|  |  | Disinfectant solutions |
|  |  | Facemask |
|  |  | Medical gown |
|  |  | Eyewear or shield |
|  |  | Gloves |

**Section four**

|  | How do you agree/disagree with the following statements? | Completely agree | Agree | Somewhat Agree | Disagree | Completely disagree |
| --- | --- | --- | --- | --- | --- | --- |
| 37 | Phone call is effective to resolve patients’ dental problems |  |  |  |  |  |
| 38 | Examine the patient for COVID-19 symptoms such as fever, cough, muscle pain, or history of contact or traveling to high-risk areas |  |  |  |  |  |
| 39 | Take COVID-19 test for patients |  |  |  |  |  |
| 40 | Reopening of dental clinics result in spreading of the virus |  |  |  |  |  |
| 41 | Possibility of continuing the dental profession by persisting coronavirus-19 |  |  |  |  |  |
| 42 | Had problems with paying basic fees |  |  |  |  |  |
| 43 | A decrease in financial income by continuing the pandemic in the future |  |  |  |  |  |
| 44 | Had symptoms of anxiety and depression during the COVID-19 pandemic |  |  |  |  |  |
| 45 | Need to talk to a psychiatrist or therapist |  |  |  |  |  |
| 46 | Follow the latest news of the COVID-19 pandemic |  |  |  |  |  |
| 47 | Latest news of COVID-19 pandemic are useful |  |  |  |  |  |
| 48 | Following the latest news is cause of my depression and anxiety |  |  |  |  |  |
| 49 | Latest guidelines of dental settings during COVID-19 are useful |  |  |  |  |  |
| 50 | The guidelines toward dental practice during COVID-19 will change in the future |  |  |  |  |  |
| 51 | PPE is effective to prevent virus transmission |  |  |  |  |  |
